# Concurrent effect of supra-threshold TENS applied over tibialis anterior muscle decreases mediolateral sway of healthy young adults

**DOI:** 10.3389/fphys.2025.1567227

**Published:** 2025-06-03

**Authors:** Marko Vidovič, Tibor Kafel, Lea Šuc, Darja Rugelj, Daša Weber

**Affiliations:** ^1^ Rehabilitation Institute, Republic of Slovenia, Ljubljana, Slovenia; ^2^ Faculty of Medicine, University of Ljubljana, Ljubljana, Slovenia; ^3^ Biomechanical Laboratory, Faculty of Health Sciences, University of Ljubljana, Ljubljana, Slovenia

**Keywords:** postural control, neuromodulation, eyes closed, healthy people, transcutaneous electrical nerve stimulation

## Abstract

**Introduction:**

Postural stability during quiet standing relies on effective sensorimotor integration. Sensory stimulation techniques, commonly applied to the lower limbs, have been used to enhance sensory input and reduce postural sway. This study investigated the immediate effects of transcutaneous electrical nerve stimulation (TENS) applied over the tibialis anterior (TA) and triceps surae (TS) muscles on postural sway in healthy young adults. A secondary aim was to compare the postural responses between the two stimulation sites.

**Methods:**

Twenty healthy volunteers (28.1 ± 3.8 years) participated in this study. Supra-threshold TENS at 100 Hz was applied over the TA and TS muscles in two separate sessions. Four postural sway variables were analyzed in the time domain: center of pressure (CoP) velocity, mediolateral and anteroposterior path lengths, and sway area. Testing was performed under both eyes-open and eyes-closed conditions.

**Results:**

TENS applied over the TA muscle significantly decreased mediolateral sway path length under eyes-closed conditions. No significant effects were observed for stimulation over the TS muscle, nor for either site under eyes-open conditions.

**Discussion:**

These findings suggest that supra-threshold TENS at 100 Hz applied over the TA muscle may improve postural stability in more demanding sensory conditions (i.e., eyes closed), with effects specifically observed in the mediolateral direction. No significant impact of TS stimulation was found under either visual condition. Further research is warranted to investigate the effects of prolonged or repeated TENS application and its potential to enhance postural control.

## 1 Introduction

Postural stability is maintained through continuous subconscious control of postural sway. During quiet stance, afferent feedback from mechanoreceptors in muscles, tendons, and joints plays a critical role in the regulation of balance (Forbes et al., 2018). In particular, muscle spindle afferents provide the most accurate and timely information regarding body position and movement relative to the supporting surface (Proske, 2006). Postural oscillations during quiet stance are closely correlated with ankle joint rotations, suggesting that sensory receptors in the ankle muscles, namely, the tibialis anterior (TA) and triceps surae (TS), provide sufficient proprioceptive input to sustain upright posture (Loram et al., 2005; Magalhães and Kohn, 2014).

To regulate postural adjustments, the central nervous system integrates afferent input from somatosensory, visual, and vestibular systems. Additionally, cutaneous input from the soles of the feet contributes substantially to somatosensory integration for postural control. In attempts to enhance somatosensory input and reduce postural sway, a variety of sensory stimuli typically applied to the lower limbs have been investigated. These include vibratory and electrical stochastic resonance stimulation (Woo et al., 2017), textured insoles (Kenny et al., 2019), and transcutaneous electrical nerve stimulation (TENS) (Dickstein et al., 2006; Laufer and Dickstein, 2007), all of which have demonstrated a stabilizing effect by reducing center of pressure (CoP) fluctuations.

Sub-sensory electrical or mechanical stimulation has been shown to attenuate postural sway when applied over the skin covering muscles involved in postural control (Magalhães and Kohn, 2014; Dickstein et al., 2006; Magalhães and Kohn, 2012; Toledo et al., 2017). These effects are generally explained through the concept that certain levels of stochastic “noise” can enhance somatosensory sensitivity, lower the sensory detection threshold, and improve the central nervous system’s responsiveness to small afferent signals (McDonnell and Ward, 2011; Paillard, 2021a).

Stabilizing effects of electrical stimulation have been reported both for sub-threshold (Magalhães and Kohn, 2014; Magalhães and Kohn, 2012; Collins et al., 2011) and supra-threshold (Dickstein et al., 2006; Laufer and Dickstein, 2007; Saadat et al., 2017; Amiridis et al., 2005; Tyson et al., 2013) intensities. An optimal sub-threshold level, where stimulation is most effective, has been described by Breen et al. (Breen et al., 2014) and Magalhães et al. (Magalhães and Kohn, 2014). However, identifying this optimal intensity is time-consuming and may not be feasible in clinical practice. Therefore, the use of conventional TENS, a widely accepted therapeutic modality, with supra-threshold sensory stimulation has gained interest as a practical alternative. Despite this, results from previous studies using TENS have been inconsistent due to variations in methodology. TENS has been used to examine postural control in diverse populations, including healthy young adults (Dickstein et al., 2006; Laufer and Dickstein, 2007), older adults (Rugelj et al., 2020), individuals with peripheral neuropathy (Saadat et al., 2017), and stroke survivors (Tyson et al., 2013). Most of these studies applied conventional TENS at 100 Hz with supra-threshold intensities. For example, Dickstein et al. (Dickstein et al., 2006), Laufer et al. (Laufer and Dickstein, 2007), and Saadat et al. (Saadat et al., 2017) used fixed-frequency TENS (100 Hz), while Rugelj et al. (Rugelj et al., 2020) used a frequency sweep from 5 to 200 Hz, and Tyson et al. (Tyson et al., 2013) used a sweep from 70 to 130 Hz.

The stimulation site (i.e., electrode placement) appears to influence outcomes. When electrodes were placed above the gastrocnemius muscle, Dickstein et al. (Dickstein et al., 2006) reported a significant reduction in average sway velocity and a trend toward reduced maximal mediolateral and anteroposterior sway velocities. In contrast, electrodes applied to the medial and lateral aspects of the posterior knee (Laufer and Dickstein, 2007) also reduced mean sway velocity, whereas Saadat et al. (Saadat et al., 2017) found no such effect in diabetic neuropathy patients with electrodes placed in the same region. Similarly, Rugelj et al. (Rugelj et al., 2020) reported no effect of either sub- or supra-threshold stimulation applied over the plantar surface of the foot and posterior shank.

It has traditionally been assumed that the TS muscle plays a dominant role in postural stabilization, acting as the primary source of proprioceptive input during standing (Nashner, 1976). More recent evidence, however, suggests that the TA muscle may provide superior proprioceptive feedback under static conditions (Giulio et al., 2009). Findings from noise stimulation studies remain mixed. Magalhães and Kohn (Magalhães and Kohn, 2014) found that imperceptible electrical noise applied individually to either the TA or TS muscle led to similar reductions in CoP velocity and sway area, suggesting that both muscles may contribute equally to postural control under such conditions. Furthermore, Dickstein et al. (Dickstein et al., 2006) reported a reduction in average sway velocity and trends toward decreased maximal mediolateral and anteroposterior sway when electrodes were placed solely above the gastrocnemius muscle.

Although many studies have applied sensory electrical stimulation to elderly or clinical populations, fewer have systematically explored its effects in healthy young adults. Dickstein et al. (2006) and Laufer and Dickstein (2007) demonstrated that supra-threshold TENS applied to the posterior legs or the lateral aspects of the knees can significantly reduce postural sway in young adults, particularly in the mediolateral direction. These studies used conventional TENS parameters (100 Hz, short pulse duration) and showed that the effects depend on the stimulation site. In a recent review, Paillard (2021b) concluded that while sensory electrical stimulation holds potential for improving postural balance, the evidence in healthy young individuals remains limited and inconsistent. Our study builds upon these findings and addresses an existing gap by comparing the effects of stimulation applied over two functionally distinct muscles TA and TS, in a healthy young population.

The novelty of the present study lies in the direct comparison of the immediate effects of supra-threshold TENS applied over the TA and TS muscles on postural sway in healthy young adults. Whereas prior research has typically examined the effects of either sub-threshold noise stimulation or supra-threshold TENS in isolation, often within clinical populations or using heterogeneous stimulation sites, no study to date has systematically compared these two muscles under standardized, clinically relevant conditions. Moreover, the use of conventional TENS parameters (100 Hz, short pulse duration) within a concurrent stimulation-and-assessment design offers new insight into muscle-specific afferent contributions to postural control.

This study aimed to investigate whether conventional TENS (100 Hz, short pulse duration), applied individually over the skin covering the belly of the TA or TS muscle, could attenuate postural sway in healthy young adults during quiet stance. A secondary aim was to compare the postural responses between the two stimulation sites. Previous studies examining the efficacy of noise stimulation over various parts of the lower extremities have reported that optimal stimulation levels exist not only for sub-threshold, but also for supra-threshold intensity (Severini and Delahunt, 2018). However, sub-sensory intensities are difficult to standardize and to analyze as a function of stimulation parameters. Therefore, we opted to use supra-threshold stimulation, defined as a level above the sensory threshold, perceived as distinct tingling, yet still below the motor threshold. We hypothesized that supra-threshold TENS would enhance postural steadiness (i.e., reduce postural sway), and that both TA and TS muscles, due to their proprioceptive roles during quiet stance, would yield similar stabilizing effects.

## 2 Materials and methods

### 2.1 Participants

Twenty healthy young volunteers participated in the study. The mean age was 28.1 ± 3.8 years. The detailed descriptive data are presented in Table 1. The inclusion criteria were no prior lower leg injuries or conditions that could affect their balance. All participants received supra-threshold stimulation of the anterior tibialis muscle and of the triceps surae muscle on two separate occasions; thus, the carry-over effect was avoided. The order of stimulation area was randomized. The study was approved by the Slovenian National Medical Ethics Committee (0120-309/2018/3). Before the measurements, all participants read the information about the testing protocol. In addition, verbal explanations were provided when necessary. Participants signed the informed consent before the measurements began.

**TABLE 1:** Descriptive data of the participants in the study (N = 20).

Variable	Mean (SD)	Min	Max
Age (years)	28.1 (3.8)	22	32
Body mass (kg)	69.2 (13.2)	50	92
Body height (cm)	173.4 (9.3)	160	192
BMI (kg/m^2^)	22.9 (2.4)	18	27

SD, standard deviation.

The sample size (N = 20) was determined based on the study by Rugelj et al. (2020), which employed a similar within-subject design to assess the effects of sub- and supra-threshold TENS on postural sway. Due to the methodological similarities, including Stabilometric protocols and stimulation parameters, we considered this sample size appropriate for detecting meaningful effects. Furthermore, a *post hoc* power analysis using an estimated medium-to-large effect size (f = 0.35) indicated that the chosen sample size provided sufficient statistical power to detect significant within-subject differences in the primary outcome measures.

### 2.2 Procedure

#### 2.2.1 Transcutaneous electrical nerve stimulation (TENS)

For electrical stimulation, a Sono Plus 6920 (Enraf Nonius, the Netherlands) TENS device was used. We used self-adhesive electrodes (9 × 5 cm Axelgaard PALS, Axelgaard Manufacturing Co. Ltd., CA, United States). To stimulate the area above the TA muscle, electrodes were placed above the ankle (−), and the anterior shank just below the knee (+) of both legs. To stimulate the area above the TS muscle, electrodes were placed over the Achilles tendon (−) and the medial and lateral head of the gastrocnemius as well as of both legs.

For better electrical conductivity before the electrode application, the skin was wiped with alcohol pads. The biphasic current was set to a 200 μs pulse duration and a frequency of 100 Hz. The TENS current was increased gradually until the subjects reported the first sensation. The current was then increased by 10%. The mean currents for both muscle groups are reported in Table 2. When the subjects changed their position from sitting to standing position on the force plate, the sensation of TENS was rechecked and adjusted to a supra-threshold value if necessary.

**TABLE 2:** The current intensities during the supra-threshold stimulation of the area above the tibialis anterior and gastrocnemius muscles.

Muscle	TENS (mA) left foot			TENS (mA) right foot		
	Mean (SD)	Min	Max	Mean SD	Min	Max
Tibialis anterior	13.4 (4.3)	6.0	20.4	12.9 (4.8)	3.5	21.3
Triceps surae	10.3 (3.8)	3.3	20.5	10.5 (4.2)	3.9	20.9

mA, milliamperes; SD, standard deviation.

#### 2.2.2 Stabilometric measurements

For data acquisition, the force platform Kistler 9286AA (Winterthur, Switzerland) was used, along with the corresponding data acquisition software BioWare. With the force platform, CoP movement during an upright stance was measured. Data acquisition lasted 60 s at a 200 Hz sampling rate. Data analyses were conducted offline using StabDat-V3.1 software (Author anonymous, 2008), which is a web-based application that was developed for calculating the time domain and fractal dimensions of Stabilometric signals. The data were filtered with Gaussian averaging over three adjacent points. Four sway parameters in the time domain were chosen for the analysis of postural sway: (Forbes et al., 2018): CoP velocity, (Proske, 2006), mediolateral and (Loram et al., 2005) anteroposterior path lengths, (Magalhães and Kohn, 2014), sway area calculated as the best area outline represented by the first 20 Fourier coefficients (FAO) as described elsewhere (Rugelj and Sevšek, 2011).

Participants were instructed to stand on the force platform and be as still as possible, with their feet close together, barefoot. Arms were crossed over the chest while the head was held upright, looking ahead to a point at eye height 2 m away. The measurements were conducted in a quiet, controlled environment to minimize auditory distractions and ensure consistency across conditions. If the participants moved their feet or arms from the required position or opened their eyes in the eyes-closed experiment, the measuring procedure was immediately interrupted. The participants were allowed to rest in a sitting position for at least 60 s between the measurements.

Postural sway was measured under four different conditions: eyes open or closed, with or without supra-threshold TENS applied over the TA and the TS muscle. The order of the chosen muscle was randomized with the function RAND in Excel (MS Excel, Redmond, Washington, United States).

### 2.3 Statistical analysis

For statistical analysis, the Statistical Package for Social Sciences (SPSS 26, SPSS Inc., Chicago, IL, United States) was used. For identifying the effects of the vision conditions and TENS on the postural sway-dependent variables, a 2 × 2 repeated measures analysis of variance (ANOVA) was performed. A paired sample t-test followed significant ANOVA findings. The significance level was set at p < 0.05.

## 3 Results

### 3.1 TENS applied over the tibial anterior muscle

Normality of data distribution was assessed using the Shapiro-Wilk test, confirming normal distribution for all variables. Therefore, parametric tests were used. A 2 × 2 repeated-measures ANOVA (vision: eyes open vs eyes closed; stimulation: with vs without supra-threshold TENS) was performed.

A significant main effect of vision was observed across all four postural sway variables: mean CoP velocity (F (1,18) = 41.64, p < 0.001), mediolateral (ML) path length (F (1,18) = 50.42, p < 0.001), anteroposterior (AP) path length (F (1,18) = 20.40, p < 0.001), and sway area (F (1,18) = 5.82, p = 0.026), confirming that visual input significantly influenced postural stability.

The main effect of TENS was statistically significant only for ML path length (F (1,18) = 6.47, p = 0.020), with a partial eta squared (η^2^_p_) of 0.26, indicating a large effect size. No significant effects were found for mean CoP velocity (F (1,18) = 1.38, p = 0.255), AP path length (F (1,18) = 0.11, p = 0.750), or sway area (F (1,18) = 0.73, p = 0.404). This suggests that supra-threshold TENS applied over the TA muscle selectively reduced sway in the mediolateral direction.

The interaction effect between vision and TENS stimulation was not significant for any variable: mean CoP velocity (F (1,18) = 0.55, p = 0.467), ML path length (F (1,18) = 0.94, p = 0.345), AP path length (F (1,18) = 0.27, p = 0.613), and sway area (F (1,18) = 0.02, p = 0.879). Therefore, no vision-by-stimulation interaction was observed.

A paired-samples t-test further confirmed a significant reduction in ML path length under eyes-closed conditions (t = 2.214, p = 0.035). Mean CoP velocity approached significance (p = 0.070) in the same condition.

Average percentage changes in postural sway variables under the eyes-closed condition indicated an 8.8% reduction in ML path length and a 6.1% reduction in mean CoP velocity when TENS was applied. Detailed results are presented in Tables 3, 4.

**TABLE 3:** The mean values of the four postural sway variables in eyes-open and eyes-closed conditions for the Tibialis anterior muscle application.

Measurement condition	Mean velocity (cm/s); (SD)	ML path (cm) (SD)	AP path (cm) (SD)	Sway area PCA (cm^2^); (SD)
Open eyes	1.61 (0.42)	61.9 (13.5)	60.59 (22.83)	3.35 (1.66)
Open eyes with TENS	1.6 (0.46)	58.98 (13.38)	61.69 (25.80)	3.14 (1.45)
Closed eyes	2.27 (0.48)	89.58 (19.47)	83.13 (21.57)	4.38 (2.75)
Closed eyes with TENS	2.11 (0.47)^#^	80.91 (20.77)*	79.28 (22,48)	4.07 (1.79)

ML, medio-lateral; AP, anteroposterior; SD, standard deviation, *p < 0.05, #p < 0.1.

**TABLE 4:** Percentage change for the individual postural sway variables between TENS and no-TENS conditions in eyes-open and eyes-closed conditions for the Tibialis anterior muscle application.

Measurement condition	Mean velocity (%); (SD)	ML path (%) (SD)	AP path (%) (SD)	Sway area (%); (SD)
Eyes open with TENS	5.35 (39.22)	−1.20 (27.44)	15.99 (61.31)	17.27 (75.53)
Eyes closed with TENS	−6.08 (15.51)^#^	−8.82 (18.15)*	−3.70 (14.63)	11.85 (57.42)

ML, medio-lateral; AP, anteroposterior; SD, standard deviation, *p < 0.05, #p < 0.1.

### 3.2 TENS applied over the triceps surae muscle group

A 2 × 2 repeated-measures ANOVA (vision: eyes open vs eyes closed; stimulation: with vs without supra-threshold TENS) was conducted to compare postural sway variables across four experimental conditions involving stimulation over the gastrocnemius muscle.

A significant main effect of vision was found for all analyzed postural sway variables, except sway area: mean CoP velocity (F (1,18) = 21.32, p < 0.001), mediolateral path length (F (1,18) = 46.44, p < 0.001), and anteroposterior path length (F (1,18) = 7.68, p < 0.001), indicating that visual input significantly influenced sway behavior. The effect on sway area was not significant (F (1,18) = 2.31, p = 0.145).

The main effect of TENS was not significant for any of the four sway variables: mean CoP velocity (F (1,18) = 0.10, p = 0.762), mediolateral path length (F (1,18) = 1.55, p = 0.228), anteroposterior path length (F (1,18) = 0.04, p = 0.852), and sway area (F (1,18) = 0.01, p = 0.934). This indicates that stimulation over the TS muscle had no measurable effect on postural sway under the tested conditions.

Likewise, the interaction between vision and TENS stimulation was not significant for any sway variable: mean CoP velocity (F (1,18) = 1.41, p = 0.250), mediolateral path length (F (1,18) = 0.86, p = 0.364), anteroposterior path length (F (1,18) = 1.40, p = 0.251), and sway area (F (1,18) = 0.95, p = 0.343). Thus, no interaction effects were observed for the TS condition.

The average percentage change showed a consistent but non-significant decrease in postural sway variables in the eyes-closed condition, with a 6.2% reduction in mediolateral path length and a 5.7% reduction in CoP velocity. Detailed results for all sway parameters and percentage changes are presented in Tables 5, 6.

**TABLE 5:** The mean values of the four postural sway variables in eyes-open and eyes-closed conditions for the Triceps surae muscle application.

Measurement condition	Mean velocity (cm/s); (SD)	ML path (cm) (SD)	AP path (cm) (SD)	Sway area (cm^2^); (SD)
Eyes open	1.55 (0.43)	60.29 (12.47)	57.88 (23.35)	3.52 (1.64)
Eyes open with TENS	1.67 (0.64)	59.63 (16.41)	66.53 (36.61)	3.15 (1.68)
Eyes closed	2.24 (0.59)	88.74 (22.76)	82.16 (26.05)	3.68 (1.72)
Eyes closed with TENS	2.06 (0.56)	80.88 (20.87)	75.65 (27.01)	4.09 (2.43)

ML, medio-lateral; AP, anteroposterior; SD, standard deviation.

**TABLE 6:** Percentage change for the individual postural sway variables during TENS application in eyes-open and eyes-closed conditions for the Triceps surae application.

Measurement condition	Mean velocity (%); (SD)	ML path (%) (SD)	AP path (%) (SD)	Sway area (%); (SD)
Eyes open with TENS	17.87 (58.51)	4.02 (37.76)	35.05 (90.50)	6.25 (57.97)
Eyes closed with TENS	−5.66 (21.36)	−6.22 (21,25)	−5.64 (22.52)	20.29 (59.71)

ML, medio-lateral; AP, anteroposterior; SD, standard deviation.

## 4 Discussion

Several somatosensory cues have been proposed to improve the performance of the postural control system. The purpose of the present study was to investigate the immediate, concurrent effect of supra-threshold TENS applied over the skin covering the TA and TS muscles, compared to no stimulation. The study also aimed to compare the effects between the two stimulation sites. To our knowledge, this was the first study to compare the effects of supra-threshold TENS over the TA and TS muscles under two different vision conditions. The results showed that TENS applied over the TA muscle reduced postural sway in the ML direction by 8.8% under eyes-closed conditions, while stimulation over the TS muscle produced no significant immediate effects.

The rationale for using supra-threshold TENS in this context did not rely on the stochastic resonance mechanism. The target was not peripheral membrane fluctuations (McDonnell and Ward, 2011), but rather the integration of incoming afferent signals via Ia, Ib, and II fibers and their effects on spinal and supraspinal excitability (Paillard, 2021a; Veldman et al., 2014), including potential modulation of corticospinal activity (Kenny et al., 2019) and enhanced attention toward the stimulated region. An attentional or arousal mechanism may have contributed to increased postural stability. Electrical stimuli at 100 Hz applied over muscle tissue elicit action potentials in both cutaneous and muscle afferents. Low-intensity activation of sensory axons can modify motoneuron excitability, leading to enhanced muscle activation (McDonnell and Ward, 2011). This enhanced sensory feedback may contribute to improved muscle force control (Collins et al., 2002; Blouin et al., 2009), suggesting that the added afferent input provided by TENS may induce sensory re-weighting during postural regulation.

Stimulation over the TA, but not over the TS, led to a significant reduction in postural sway under eyes-closed conditions. This differs from Magalhães and Kohn (2014), who reported reduced postural sway regardless of whether TA, TS, or both muscles were stimulated. However, a direct comparison may not be appropriate, as their study used stochastic sub-threshold noise, whereas our study employed regular, supra-threshold TENS. Both the stimulation intensity and the regularity of pulses likely influence the underlying mechanisms by which sensory input modulates postural control.

The differential responses between TA and TS may be explained by their functional roles during standing. The TS muscle has traditionally been considered the main source of proprioceptive input during quiet stance (Nashner, 1976), but more recent evidence suggests that the TA muscle may have a superior role (Giulio et al., 2009). Our findings support this notion, as additional afferent input from the skin overlying the active TA muscle, possibly engaging Ia and II afferents, appears to have been integrated into the sensory processing for postural control. In contrast, stimulation over the skin above the relatively passive TS muscle (Giulio et al., 2009) did not elicit similar effects.

The ability to control force fluctuations during postural muscle contractions is also a key contributor to postural steadiness (Davis et al., 2020). TENS applied over muscle tissue has been shown to enhance muscle steadiness (Mani et al., 2018), increase muscle activation (Gabler et al., 2016), and reduce fatigue (Enoka et al., 2020). These neuromuscular effects may also contribute to reduced postural sway when TENS is applied over the TA. Interestingly, the stabilizing effect was only evident under eyes-closed conditions, suggesting that participants were able to re-weight the added sensory input more effectively in challenging sensory contexts. This finding aligns with earlier studies showing that both sub-sensory stimulation (Toledo et al., 2017) and light touch (Jeka and Lackner, 1994) are more effective in reducing sway when visual information is absent.

Although previous studies have typically reported effects of TA stimulation on AP sway variables, our results showed a significant reduction only in the ML direction. This might be because ML sway is more dependent on rapid sensory integration and compensatory mechanisms, especially under sensory-deprived conditions. While the TA primarily contributes to sagittal plane control as an ankle dorsiflexor, supra-threshold stimulation may have affected central mechanisms in a direction-specific manner.

Additionally, CoP variables vary in their sensitivity to different types of sensory interventions. As highlighted by Paillard (2021b), not all CoP metrics equally reflect changes in postural control strategies. In our study, the significant reduction in ML path length despite no changes in CoP velocity, AP path, or sway area suggests that ML sway might be more sensitive to the type of afferent input provided by TENS under eyes-closed conditions. This does not imply that other aspects of postural control were unaffected, but rather that conventional time-domain measures may lack the resolution to detect them.

Non-linear analyses, such as sample entropy and fractal dimension, could potentially offer deeper insight into how TENS influences the structural variability and complexity of postural sway. These methods were not included in the present study, but future research should consider their use to detect subtle modulations in postural control that may not be captured by linear sway measures.

Electrode placement is another important factor that may explain discrepancies across studies. Prior work has shown that stimulation on the posterior shank can improve postural stability (Dickstein et al., 2006), whereas stimulation on the posterior knee (Saadat et al., 2017), sole, or posterior shank had no effect (Rugelj et al., 2020). The exact anatomical site and orientation of electrodes likely influence both afferent recruitment and the direction of postural adaptation.

A limitation of this study is the relatively small sample size of young, healthy individuals and the use of only one TENS protocol. Although the findings may not be generalizable, they are promising in identifying potentially clinically relevant effects of TENS on postural steadiness in populations with impaired sensory input, such as older adults or individuals with neurological conditions. Future studies should aim to determine optimal combinations of stimulation parameters and locations to maximize neuromodulatory effects. For instance, varying pulse widths can influence habituation rates (Tyson et al., 2013; Rugelj et al., 2020), and electrode orientation (parallel vs perpendicular to nerve fibers) can alter current flow and neural response. Session duration may also play a role, with different effects expected during immediate application (concurrent stimulation) versus prolonged pre-activation (Paillard, 2021a).

The potential benefits of TENS for enhancing balance and mobility, particularly in individuals after stroke (Tyson et al., 2013), support its integration into functional rehabilitation programs. Future research should explore longer-term application and training effects, especially in populations with high fall risk, such as elderly individuals and those with neurological deficits, where 30%–50% experience at least one fall per year with possible severe consequences.

## 5 Conclusion

Based on the results of this study, we conclude that supra-threshold TENS applied at 100 Hz over the TA muscle has an immediate stabilizing effect on postural sway, whereas stimulation over the triceps surae TS muscle yields less effect. Healthy young adults appear to integrate the additional afferent input provided by TENS into their postural control only under more challenging sensory conditions, such as standing with eyes closed. Regardless of the precise physiological mechanisms underlying this effect, supra-threshold TENS shows promise as a tool to enhance postural stability and may represent a useful adjunct to balance training interventions.

## Data Availability

The raw data supporting the conclusions of this article will be made available by the authors, without undue reservation.
